# A Rare Case of a Pedunculated Lipoma in the Perianal Region: A 20-Year Journey

**DOI:** 10.7759/cureus.61304

**Published:** 2024-05-29

**Authors:** Sai Goutham Rekavari, Chanrashekhar Mahakalkar

**Affiliations:** 1 General Surgery, Jawaharlal Nehru Medical College, Datta Meghe Institute of Higher Education and Research, Wardha, IND

**Keywords:** histopathological examination, lipofibroma, surgical excision, pedunculated, perianal, lipoma

## Abstract

Lipomas are common benign soft tissue tumors, typically presenting as painless, slow-growing masses of mature adipose tissue. However, their occurrence as pedunculated lesions in the perianal region is rare. We present a case of a 70-year-old male with a 20-year history of a painless, cosmetically concerning mass in the perianal region. Clinical examination and ultrasonographic findings were consistent with a pedunculated lipoma. Surgical excision was performed successfully, and histopathological examination confirmed the diagnosis of lipofibroma. This case highlights the importance of considering unusual presentations of lipomas in the differential diagnosis of perianal masses. It emphasizes the role of surgical excision for symptomatic or cosmetically concerning lesions. Long-term follow-up is essential to monitor for recurrence and ensure optimal patient outcomes.

## Introduction

Lipomas are the most common benign soft tissue tumors, composed primarily of mature adipocytes. They can occur in any body part but are most frequently found in subcutaneous tissues. The incidence of lipomas increases with age, and they are typically diagnosed in middle-aged and older adults [1]. Although lipomas are generally asymptomatic and slow-growing, their presentation can vary widely depending on their size, location, and whether they exert pressure on adjacent structures [2]. The perianal region is an unusual site for lipomas. When they do occur in this region, they can present unique diagnostic and management challenges. Perianal lipomas are rarely reported in the literature, and their occurrence in a pedunculated form is even more uncommon. Pedunculated lipomas are characterized by a stalk-like attachment to the underlying tissue, which can complicate both the diagnosis and the surgical approach [3].

Imaging studies, particularly ultrasonography, are crucial in diagnosing soft tissue tumors. Ultrasonography can help delineate the lesion's characteristics, including its echogenicity and vascularity, which are important for distinguishing lipomas from other soft tissue masses [4]. Doppler ultrasound, in particular, is useful for assessing the vascularity of the lesion, which is typically absent in lipomas, aiding in their identification as benign tumors [5]. Surgical excision remains the treatment for symptomatic lipomas or those causing cosmetic concerns. The procedure involves removing the entire lesion, including its capsule, to prevent recurrence. Histopathological examination post-excision is essential to confirm the diagnosis and rule out malignancy [6]. Regular follow-up is recommended to monitor for any signs of recurrence and ensure the patient's ongoing well-being [7]. This case report presents a unique instance of a pedunculated lipoma in the perianal region of a 70-year-old male, highlighting the clinical presentation, diagnostic workup, and successful surgical management of this rare condition.

## Case presentation

**Figure 1:**
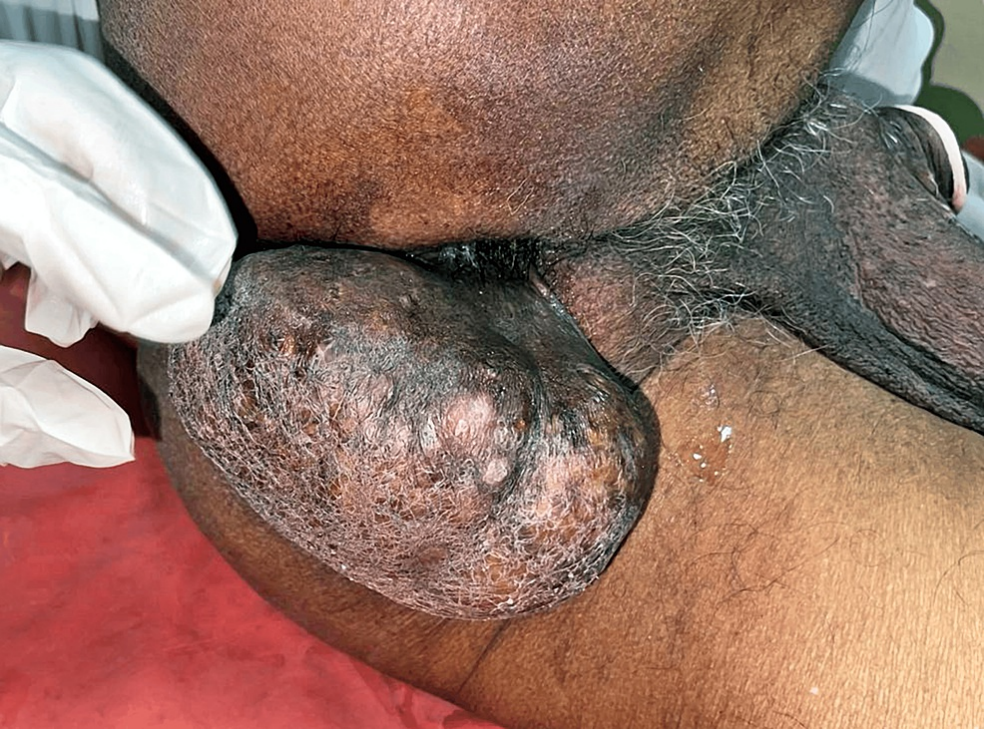
A soft, solitary, pedunculated swelling measuring approximately 7x7x6 cm on clinical examination was identified proximally to the perianal region

A 70-year-old male presented to our outpatient department with a long-standing history of a mass in the perianal region. The patient reported that the mass had been present for approximately 20 years and had grown slowly. Despite its size, the mass was painless, and there were no associated symptoms such as altered bowel habits or signs of inflammation. However, its considerable size and location had become a cosmetic concern for the patient. A soft, solitary, pedunculated swelling measuring approximately 7x7x6 cm on clinical examination was identified proximally to the perianal region (Figure 1). The mass was non-tender, showed no signs of inflammation, and had no apparent connection to the anal canal.

To evaluate the mass further, an ultrasonographic examination of the gluteal and perianal regions was performed. The imaging revealed a well-defined, pedunculated, isoechoic lesion within the subcutaneous plane. Notably, no vascularity was observed on the Doppler ultrasound, which supported the benign nature of the lesion. Given the clinical and imaging findings, a diagnosis of a pedunculated lipoma was considered, and the patient was advised to undergo surgical excision of the mass.

**Figure 2:**
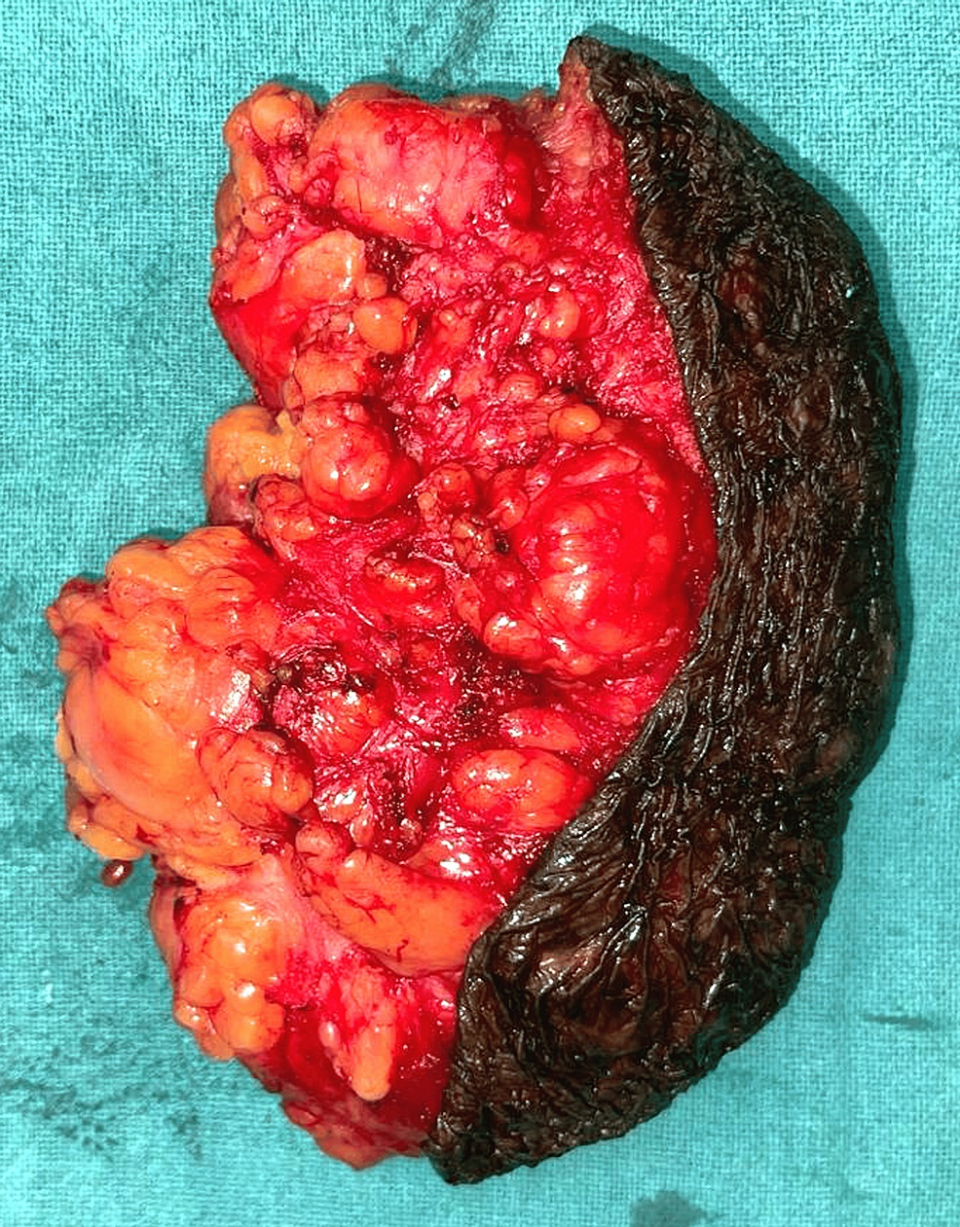
A lipomatous tissue was carefully excised, and hemostasis was achieved before performing a primary wound closure

The surgical procedure was carried out under general anesthesia. An elliptical incision was made around the mass's pedicle, and the dissection was deepened to reach the subcutaneous plane. The lipomatous tissue was carefully excised, and hemostasis was achieved before performing a primary wound closure (Figure 2). The patient tolerated the procedure well and had an uneventful postoperative course. He was discharged following suture removal and scheduled for regular follow-up visits. During the 24-month follow-up, the patient showed no signs of recurrence and remained asymptomatic.

**Figure 3:**
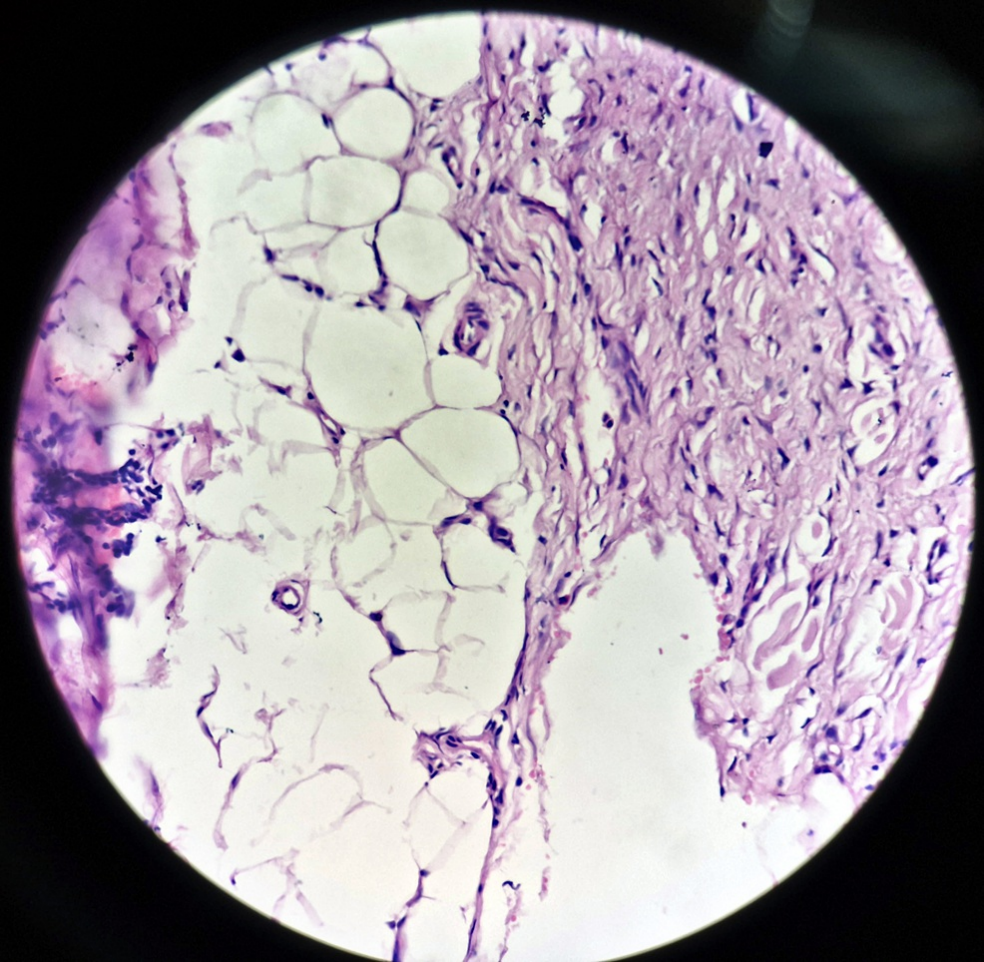
The excised mass confirmed the diagnosis of lipofibroma, a lipoma variant characterized by fibrous tissue within the adipose tissue

Histopathological examination of the excised mass confirmed the diagnosis of lipofibroma, a lipoma variant characterized by fibrous tissue within the adipose tissue (Figure 3). Despite its unusual presentation in this region, this case underscores the importance of considering lipoma in diagnosing perianal masses. Surgical excision provided a definitive diagnosis and alleviated the patient's cosmetic concerns. Regular follow-up is essential to monitor for any signs of recurrence, ensuring long-term patient satisfaction and health.

## Discussion

Lipomas are the most common benign soft tissue tumors, typically presenting as slow-growing, painless masses of mature adipose tissue. They can occur anywhere in the body where fat is present but are most frequently found in the subcutaneous tissues of the trunk and proximal extremities. While lipomas are generally straightforward in their presentation, the occurrence of a pedunculated lipoma in the perianal region, as seen in this case, is relatively rare and poses unique clinical challenges. The patient in this report presented with a large, pedunculated mass in the perianal region that had been present for 20 years. This protracted history, coupled with the absence of symptoms such as pain or bowel habit changes, is typical of lipomas, which are often asymptomatic unless they reach a size or location that causes discomfort or cosmetic concerns. The clinical examination and ultrasonographic findings were consistent with a benign lesion, showing a well-defined, isoechoic mass with no vascularity on the Doppler ultrasound, which is characteristic of lipomas [4,8].

Surgical excision is the treatment for lipomas that are symptomatic, cosmetically concerning, or show any atypical features that raise suspicion for malignancy [9]. In this case, the surgical approach involved an elliptical incision around the pedicle, careful dissection to identify and remove the lipomatous tissue, and primary wound closure. This method effectively addressed the patient's cosmetic concerns and the potential for future complications associated with the mass. Histopathological examination confirmed the diagnosis of lipofibroma, a lipoma variant with a significant fibrous component. This variant is known to be less common. It can sometimes be confused with other fibrous or adipose tissue tumors, highlighting the importance of histopathological evaluation in cases of large or atypically presenting lipomas [10].

The rarity of pedunculated lipomas in the perianal region necessitates careful differential diagnosis. Other conditions that could present similarly include epidermoid cysts, dermoid cysts, pilonidal disease, and, more rarely, soft tissue sarcomas [11]. The absence of inflammatory signs and benign characteristics in ultrasonography helped narrow down the diagnosis to a lipomatous lesion in this case. Long-term follow-up is crucial for patients who have undergone excision of large lipomas to monitor for recurrence. Although lipomas rarely recur, incomplete excision or the presence of multiple lipomas can lead to recurrence [12]. This patient's regular follow-up visits have shown no signs of recurrence, indicating a successful surgical outcome.

## Conclusions

In conclusion, the presented case of a pedunculated lipoma in the perianal region underscores the importance of recognizing uncommon presentations of benign soft tissue tumors. Despite its rarity and unique location, the diagnosis was successfully established through a comprehensive approach involving clinical assessment, imaging studies, and histopathological examination. Surgical excision emerged as the definitive management strategy, effectively addressing diagnostic confirmation and the patient's cosmetic concerns. This case highlights clinicians' need to maintain a broad differential diagnosis, especially when encountering atypical manifestations of common conditions. Furthermore, long-term follow-up is crucial to monitor for recurrence and ensure optimal patient outcomes. Overall, this case contributes valuable insights to the literature on lipomas and emphasizes the significance of individualized patient care in addressing rare clinical presentations.
